# Comparative Study of the Dynamic Deformation of Pure Molybdenum at High Strain Rates and High Temperatures

**DOI:** 10.3390/ma14174847

**Published:** 2021-08-26

**Authors:** Shuai Chen, Wen-Bin Li, Xiao-Ming Wang, Wen-Jin Yao, Jiu-Peng Song, Xiang-Cao Jiang, Bin-You Yan

**Affiliations:** 1 School of Mechanical Engineering, Nanjing University of Science and Technology, Nanjing 210094, China; 202xm@163.com (X.-M.W.); njyaowj@163.com (W.-J.Y.); 2 National R&D Center for Tungsten Technology & Xiamen Tungsten Co., Ltd., Xiamen 361006, China; song.jiupeng@cxtc.com (J.-P.S.); jiang.xiangcao@cxtc.com (X.-C.J.); yan.binyou@cxtc.com (B.-Y.Y.); 3 School of Materials Science and Engineering, Xihua University, Chengdu 610039, China

**Keywords:** molybdenum, dynamic mechanical properties, high strain rate, modified Johnson–Cook constitutive model

## Abstract

To study the dynamic plastic properties of high-purity molybdenum materials at high temperature and high strain rate, we designed tests to compare the mechanical behaviour of two high-purity molybdenum materials with different purities and two with different processing deformation conditions under dynamic impact compression in the temperature range of 297–1273 K. We analysed the molybdenum materials’ sensitivities to the strain-hardening effect, strain rate-strengthening effect, and temperature-softening effect as well as the comprehensive response to the combined effect of the strain rate and temperature, the adiabatic impact process, and the microstructure at high temperature and high strain rate. Furthermore, based on a modified Johnson–Cook constitutive model, we quantitatively analysed the flow stresses in these materials. The calculation results strongly agree with the test results. Our findings indicate that the high-purity molybdenum materials show consistent sensitivity to the combined effect of strain rate and temperature regarding the dynamic plastic properties. The materials with higher purity are less sensitive to the combined effect of the strain rate and temperature, and those with less processing deformation experience more pronounced strain-hardening effects. Under high strain rate at room temperature, these materials are highly susceptible to impact embrittlement and decreases in dynamic plastic properties due to intergranular fracture in the internal microstructure. However, increasing the impact environment temperature can significantly improve their plastic properties. The higher the temperature, the better the plastic properties and the higher the impact toughness.

## 1. Introduction

Shaped charge collapse liners to EFP, JPC, and JET penetrators with ultra-high detonation pressure [1,2,3,4,5,6]. In the weapon industry, the penetrators are applied to pierce armour equipment and destroy solid targets as concrete [7,8,9,10]; the penetrators are also valuable in piercing oil exploration wells for civil application. However, due to the development of new types of armoured vehicles and the enhancement of protective capabilities, the materials and structure of liners must be redesigned to improve the penetration performance of the penetrators.

In recent years, many scholars have carried out extensive research on liner materials [11,12,13]; Li Ding [14] studied the performance of EFP formed by W alloys, Ni, U, and U alloy as high-density liner materials; in contrast, Ni materials liners have the best performance of EFP. Jacek Borkowski [15], Nengquan Duan [16] studied the penetration performance of penetrators formed by sintered copper liners and proved that liners made by powder metallurgy technology were a fully featured substitute for conventional spherical liners. Guo Wenqi [17] and Elshenawy T [18] studied the penetration ability of a shaped charge jet formed by a W-Cu liner, and the relative density of the damage element and target had a significant effect.

With high density, high acoustic velocity, and excellent ductility plasticity, molybdenum is comparable to copper on penetrators’ formation performance in the studies of Al, Ni, Cu, Mo, Ta, U, and W as liner materials [19]. However, molybdenum shows complex phenomena such as high ductile–brittle transition temperature, low-temperature toughness, and high flow stress at high temperature in application [20,21]. Although it has been used in the shaped charge liner [2,5,8,22,23,24,25,26,27,28], the research on the dynamic plastic properties of molybdenum is still not completed [29,30,31].

A large number of studies have been conducted on the plastic behaviour of molybdenum [32,33], and several constitutive models [33,34,35,36,37] and state equations [38,39,40] have been developed. Many JET-forming tests of processed molybdenum liner have found that molybdenum JETs exhibit apparent brittle behaviour [2,22,28]. It is inappropriate to consider the mechanical response of molybdenum materials in shaped charges to be elastoplastic [26] while ignoring the impacts of the strength [24] and malleability [22,28] on the molybdenum JET. Researching the dynamic plastic properties of different microstructures of molybdenum materials under a wide range of temperatures and impact strain rates [22,29,41,42,43] is significant for understanding and improving the performance of molybdenum materials under impact loading and in shaped charges.

In this paper, three kinds of molybdenum with different purity and processing technology were designed. Firstly, dynamic compression tests were carried out at high strain rates under 297–1273 K, and the comprehensive response of microstructures of molybdenum materials under high-strain rate adiabatic impact was studied. Secondly, the sensitivities of molybdenum materials to the strain-hardening effect, temperature-softening effect, strain rate-strengthening effect, and strain rate-temperature combination effect were compared. Thirdly, the MJC dynamic performance evaluation model was established to evaluate molybdenum materials’ dynamic adiabatic impact under high temperature and high strain rates. Finally, conclusions are drawn to summarise our findings.

## 2. Materials and Experimental Procedures

### 2.1. Test Material

**Figure 1:**
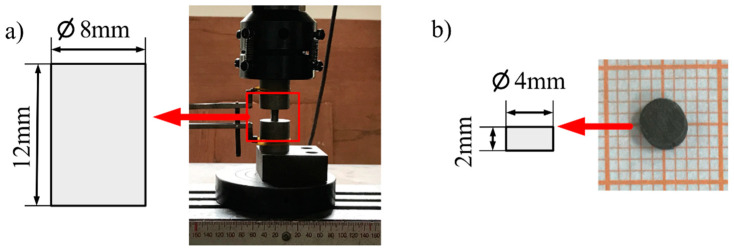
The structure diagram of test samples: (**a**) quasi-static compression sample (**b**) dynamic compression sample.

We study three types of pure molybdenum materials, two of which are high-purity powder metallurgical molybdenum materials, and the other is common pure molybdenum material for commercial use. The pure molybdenum materials were manufactured via power metallurgy (P/M) by isostatically-pressed Mo power and subsequently sintering at high temperature. The purity of high-purity molybdenum reaches 99.99%. By design, Mo-L has little deformation from powdered processing to finished product processing; Mo-H has a large amount of deformation from powder to finished product. The common commercial molybdenum material Mo-C has a purity of 99.95%. The compositions of the three molybdenum materials are listed in Table 1. For comparison purposes, mechanical test samples with dimensions of Φ8×12 mm(Φ is diameter) were prepared using the Mo-L, Mo-H, and Mo-C (Figure 1a). Mechanical test samples with dimensions of Φ4×3 mm were also prepared for SHPB experiments (Figure 1b).

### 2.2. Experimental Method

A quasi-static compression testing machine was used to obtain the true stress–strain relationship of the three pure molybdenum materials at a low strain rate. The high-temperature synchronically assembled Hopkinson dynamic compression device [44,45,46,47] was used to collect dynamic mechanical data of the three pure molybdenum materials at high temperature and high strain rate, with temperatures ranging from 297 to 1273 K. In order to ensure the accuracy of the test data, more than three repeated tests were carried out under the same deformation condition, and the stress–strain relationships of Mo-L, Mo-H, and Mo-C under different conditions were investigated.

**Figure 2:**
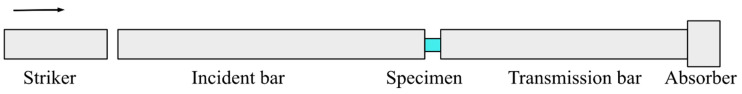
Schematic of Hopkinson dynamic compression test system with only the key components displayed.

Figure 2 shows a schematic of a conventional SHPB system. A short specimen is sandwiched between the incident bar and the transmission bar. The striker impacts the incident bar so that a stress pulse travels along the incident bar. When the stress pulse reaches the bar and specimen interface, part of it ($\epsilon_{R}$) is reflected, and part of it ($\epsilon_{T}$) is transmitted to the transmission bar. According to the basic assumption of the Hopkinson dynamic compression test, the two-wave approach [48] can be used to obtain the expressions of stress, strain, and strain rate, as in Formulas (1)–(3), where $A_{0}$ is the cross-sectional area of the specimen, $l_{0}$ is the original length of the specimen, $\epsilon_{T}$ is the strain of the transmitted wave propagating in the rod, $\epsilon_{R}$ is the strain of the reflected wave propagating in the rod, $A_{s}$ and $E_{0}$ are the cross-sectional area and elastic modulus of the rod, $C_{0}$ is the sound velocity of the rod material, and t is the pulse width of the reflected wave.

(1) $$\sigma =\frac{A_{s}E_{0}}{A_{0}}\epsilon_{T}$$

(2) $$\epsilon =-\frac{2C_{0}}{l_{0}}\int_{0}^{t}\epsilon_{R}d\tau$$

(3) $$\dot{\epsilon }=-\frac{2C_{0}}{l_{0}}\epsilon_{R}$$

## 3. Experimental Results and Discussion

### Metallographic Examination

The dynamic deformation tests were carried out under temperatures of 297 K, 473 K, 673 K, 873 K, 1073 K, and 1273 K with different impact velocities. The test of one temperature and one impact velocity was repeated more than three times. However, for the comparison of metallographic tests, the microstructures of the Mo-L, Mo-H, and Mo-C along the impact direction at temperatures of 297 K and 1273 K were examined.

**Figure 3:**
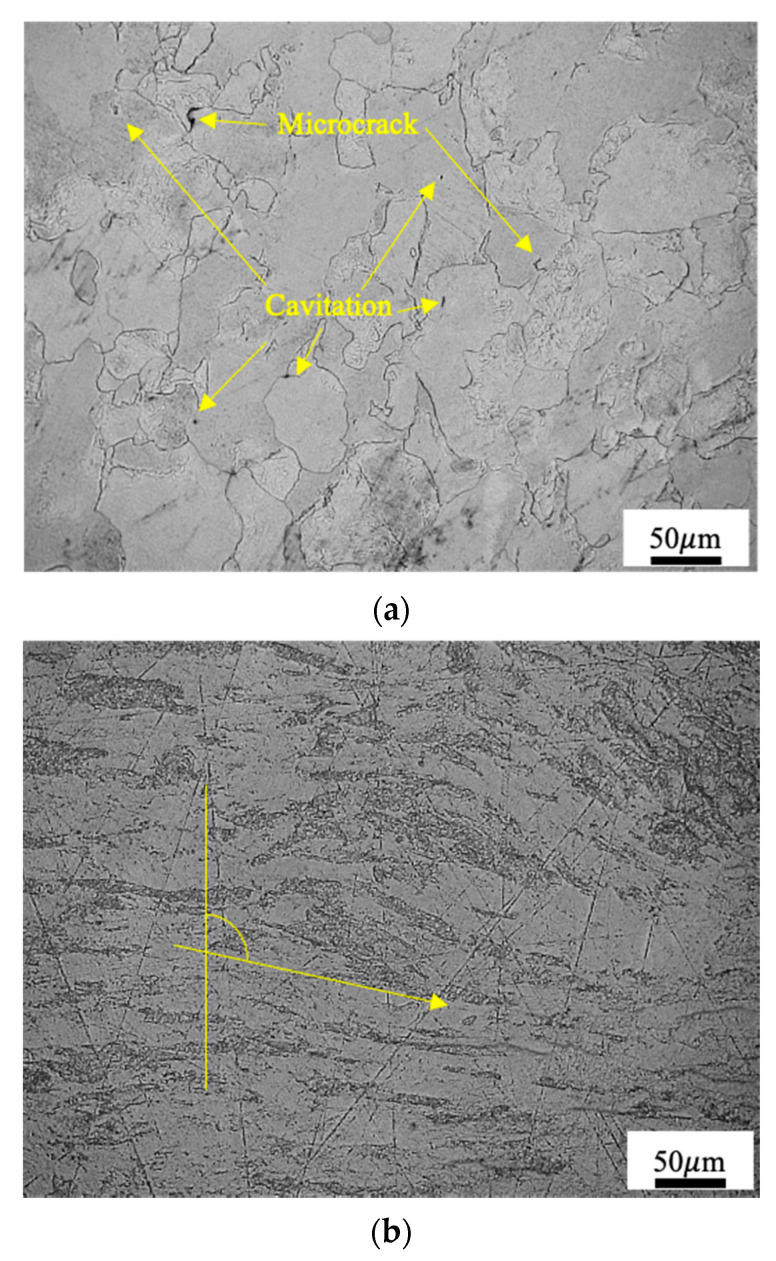
Structure of Mo-L after the impact in Hopkinson bar at (**a**) 297 K, $\dot{\epsilon }=4000/s$ and (**b**) 1273 K, $\dot{\epsilon }=6752/s$.

The microstructure of Mo-L under a strain rate of 4000/s at 297 K is shown in Figure 3a. The grains are deformed, and some have even been crushed. There are micro-cracks at the junctures of grain boundaries. The black spots inside the grains are voids that formed during the process of rapid deformation. The microstructure under a strain rate of 6752/s at 1273 K is shown in Figure 3b. The grains are compressed and elongated in the direction perpendicular to the impact direction. A large number of fibrous grains appear pointing in a certain direction, but no clear defects are found in the impacted structure.

**Figure 4:**
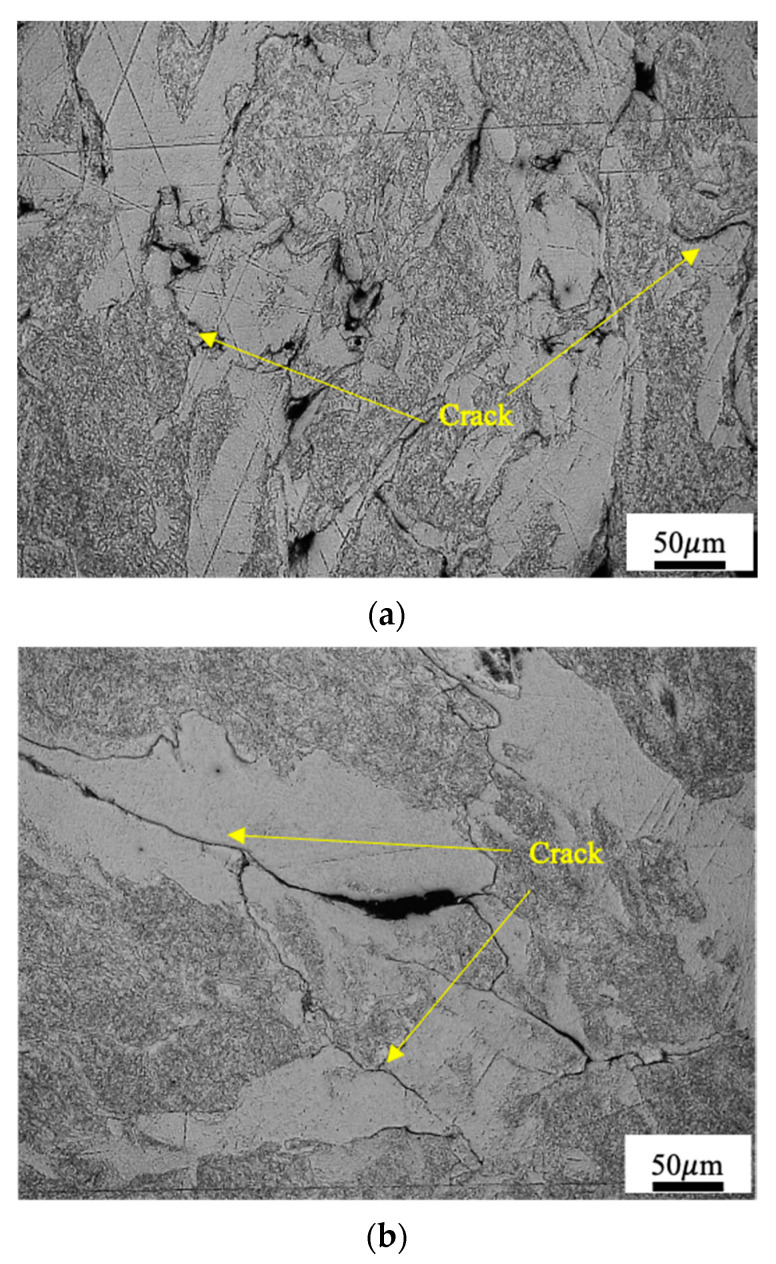
Structure of Mo-H after the impact in Hopkinson bar at (**a**) 297 K, $\dot{\epsilon }=11,000/s$ and (**b**) 1273 K, $\dot{\epsilon }=6289/s$.

The structure of Mo-H under a strain rate of 11,000/s at 297 K is shown in Figure 4a. There are many cracks along the grain boundaries distributed throughout the field of view. The grains are distorted irregularly, with noticeable small recrystallised grains inside the grains and on their edges. The structure under a strain rate of 6289/s at 1273 K is shown in Figure 4b. There are cracks passing through the grains that are obviously enlarged. The grain outlines are unclear. This finding indicates that under the impact of high temperature, recrystallisation occurs in the microstructure of the molybdenum material.

**Figure 5:**
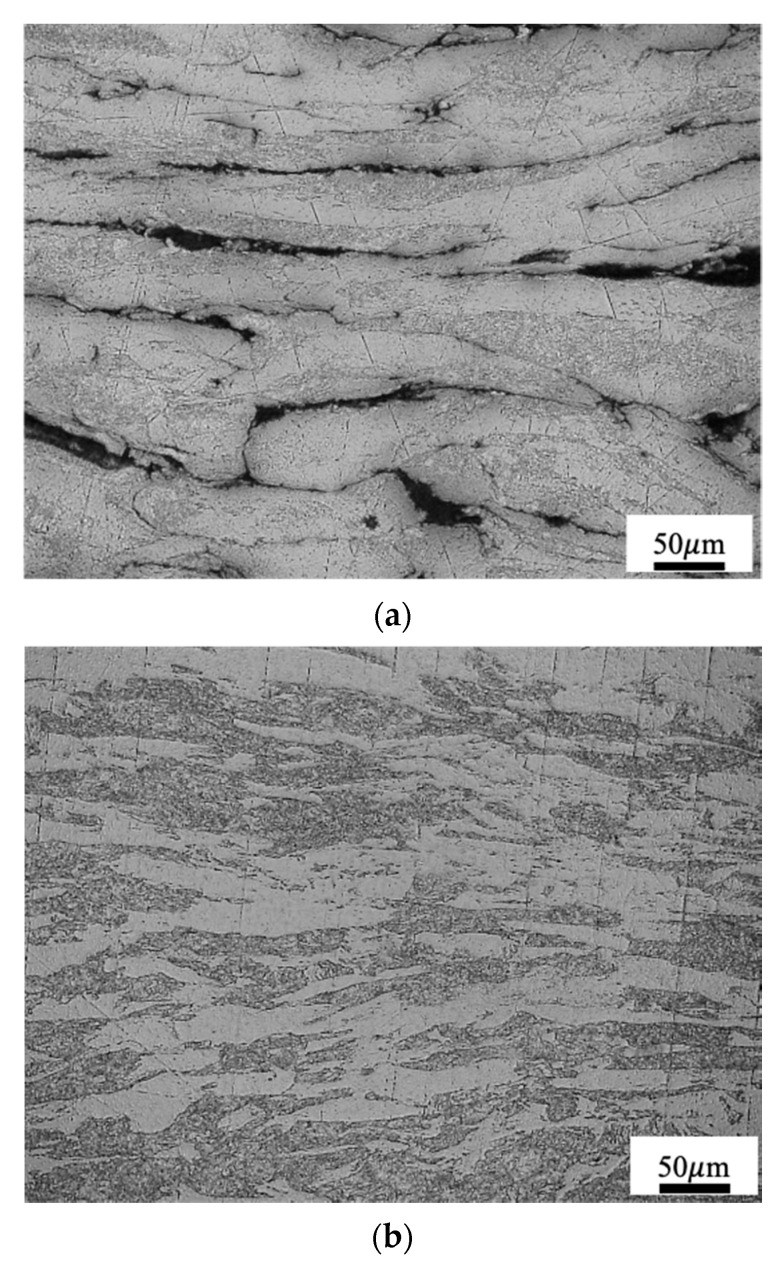
Structure of Mo-C after the impact in Hopkinson bar at (**a**) 297 K, $\dot{\epsilon }=10,180/s$ and (**b**) 1273 K, $\dot{\epsilon }=7905/s$.

The structure of Mo-C under a strain rate of 10,180/s at 297 K is shown in Figure 5a. The grains, distributed in micro-layers, show an obvious sheet texture. There are clear fractures on the boundaries of the grains in the micro-layers. The structure under a strain rate of 7905/s at 1273 K is shown in Figure 5b. Obviously, the grains are refined and become fibrous. Although the grains are severely compressed and elongated, they are evenly distributed, with no defects such as micro-cracks or voids.

## 4. Mechanical Property Evaluation

### 4.1. Stress–Strain Relationship

**Figure 6:**
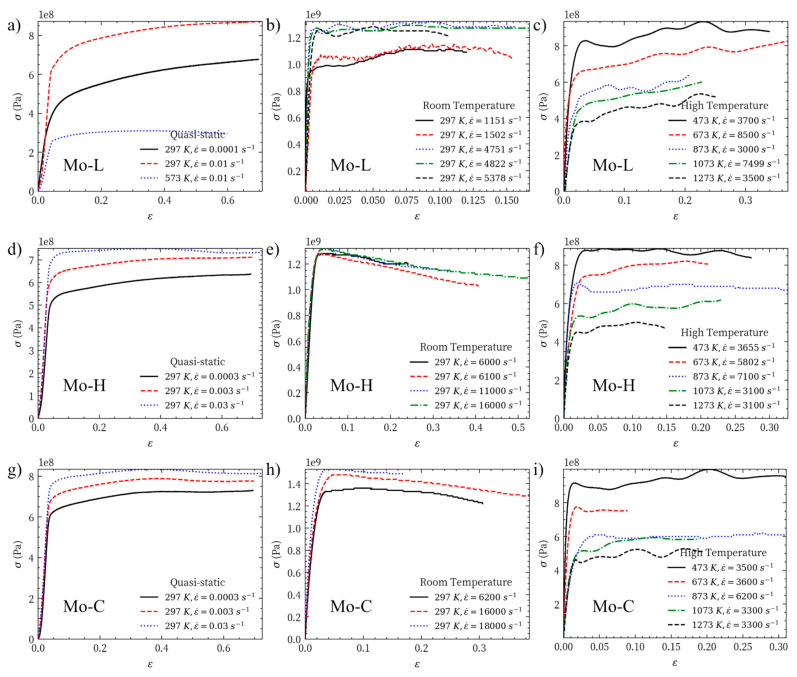
Mechanical properties of (**a**) Mo-L, quasi-static compression (**b**) Mo-L, room temperature dynamic impact (**c**) Mo-L, high temperature dynamic impact (**d**) Mo-H, quasi-static compression (**e**) Mo-H, room temperature dynamic impact (**f**) Mo-H, high temperature dynamic impact (**g**) Mo-C, quasi-static compression (**h**) Mo-C, room temperature dynamic impact (**i**) Mo-C, high temperature dynamic impact.

The true stress–strain test data of Mo-L, Mo-H, and Mo-C at different temperatures and strain rates are shown in Figure 6. In particular, Figure 6a,d,g show the true stress–strain curves under quasi-static compression; Figure 6b,e,h provide the true stress–strain curves at room temperature and high strain rate; Figure 6c,f,i present the mechanical response under the dynamic impact every 200 K in the temperature range of 473 to 1273 K. According to the flow stresses of Mo-L, Mo-H, and Mo-C in this temperature range, Mo-L shows evident strain hardening at different temperatures; Mo-C has the least strain hardening at different temperatures; and Mo-H shows strain hardening at temperatures of 673 K and 1073 K but has ideal elastoplasticity at other temperatures.

### 4.2. Sensitivity to the Strain Rate

Mo-L, Mo-H, and Mo-C have different responses at different temperatures and strain rates. According to the responses of the three molybdenum materials’ flow stresses to strain, temperature, and strain rate, we study the sensitivity of the three molybdenum materials to strain rate at room temperature (297 K). The yield strengths of the three molybdenum materials at different strain rates are shown in Table 2.

**Figure 7:**
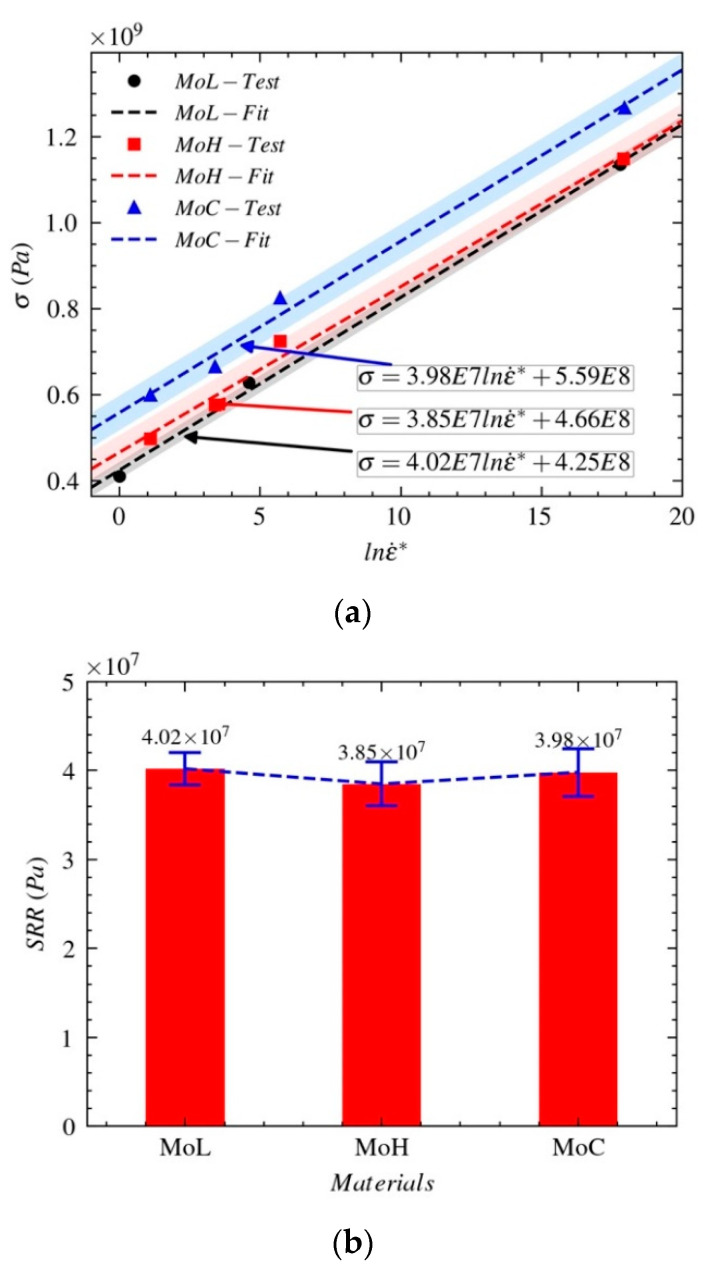
Stress–strain rate relationships of the three molybdenum materials: (**a**) stress–strain rate relationships (**b**) strain rate ratio.

When $\dot{\epsilon_{0}}=0.0001{\;s}^{-1}$, we have the dimensionless strain rate $\dot{\epsilon^{*}}=\dot{\epsilon }/\dot{\epsilon_{0}}$. The relationships between the yield strength $\sigma_{st}$ and the dimensionless strain rate $ln\dot{\epsilon^{*}}$ of Mo-L, Mo-H, and Mo-C at different temperatures and loading rates are shown in Figure 7. In particular, both $A_{d}$ and $C_{d}$ are determined.

(4) $$\sigma_{\mathrm{st}}=C_{d}ln\dot{\epsilon^{*}}+A_{d}$$

The strain rate-strengthening effect sensitivity coefficient (strain rate ratio, SRR) in Figure 7b is obtained by determining the slope of the relationship between the true yield strength with the strain rate. The SRR can be used to evaluate the strain rate-strengthening effect of the material with load. The larger the SRR is, the more obvious the strain rate-strengthening effect during the process of dynamic deformation. As shown in Figure 7b, the sensitivities of the three molybdenum materials to strain rate are ordered as follows: Mo-L > Mo-C > Mo-H.

### 4.3. Sensitivity to Strain

Based on the result of the quasi-static compression test at room temperature (297 K), we study how the flow stress σ of Mo-L, Mo-H, and Mo-C varies with the plastic strain $\epsilon_{p}$. The equivalent flow stress $\ln\left(\sigma -\sigma_{st}\right)$ is shown in Formula (5), where $\sigma_{st}$ is the yield strength and both $B_{d}$ and n are to be determined.

(5) $$ln\left(\sigma -\sigma_{st}\right)=lnB_{d}+nln\epsilon_{p}\;$$

**Figure 8:**
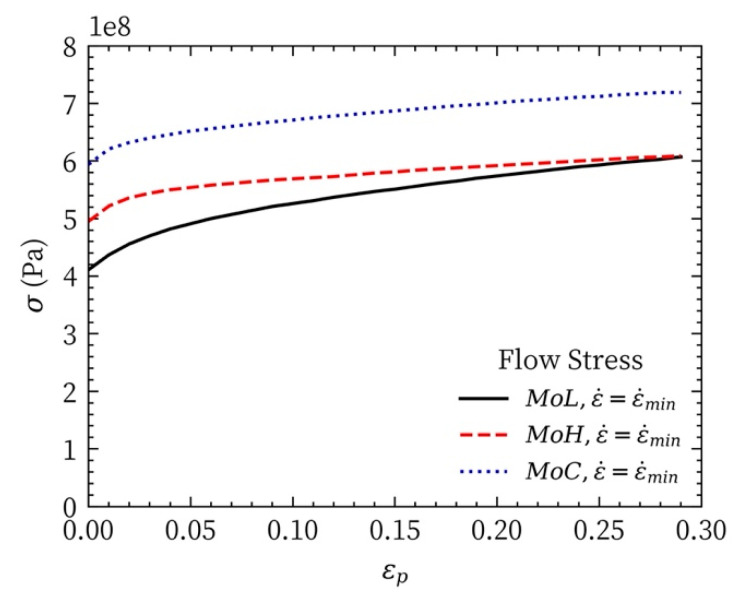
Flow stresses of the three molybdenum materials.

**Figure 9:**
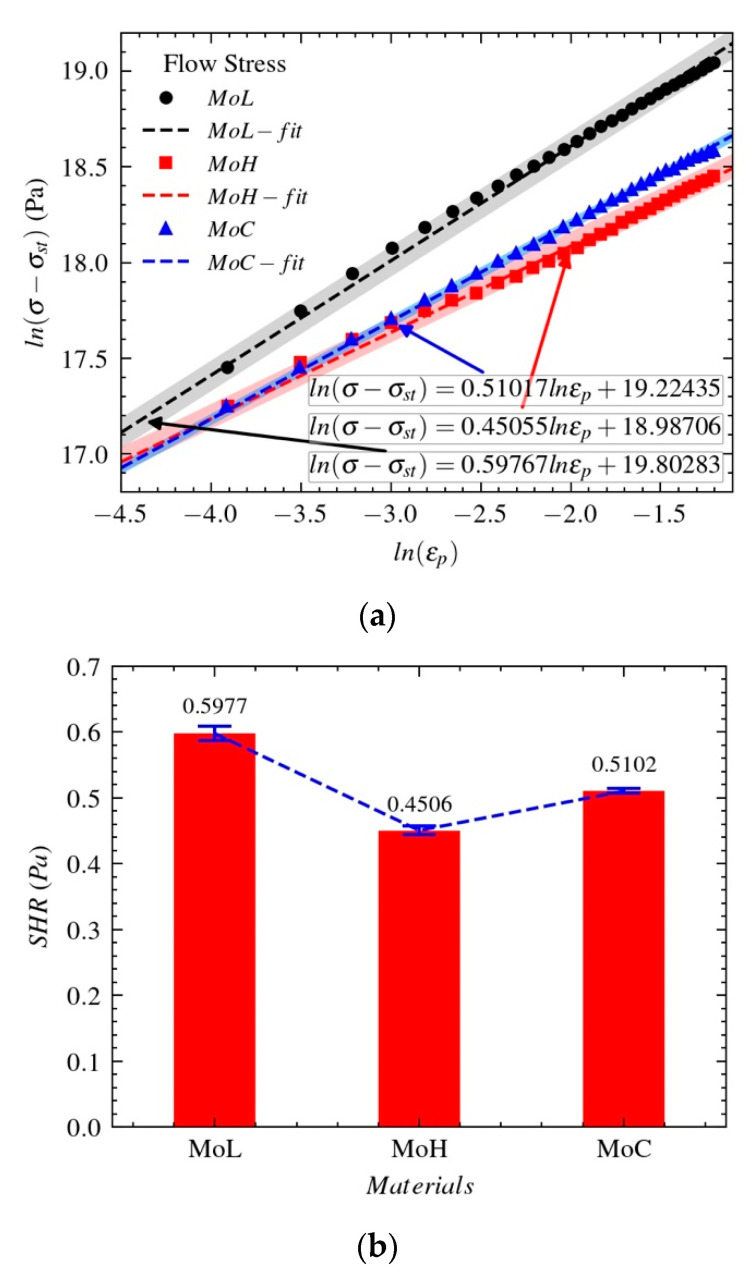
Sensitivities of the three molybdenum materials to the flow stress: (**a**) stress–strain relationships (**b**) strain-hardening ratio.

The strain-hardening characteristics as the flow stresses of Mo-L, Mo-H, and Mo-C vary with the plastic strain are shown in Figure 8. Figure 9a shows how the equivalent flow stress $\ln\left(\sigma -\sigma_{st}\right)$ varies with the plastic strain $ln\epsilon_{p}$.

The strain-hardening ratio (SHR) in Figure 9b is obtained by determining the slope of the relationship between the true stress and plastic strain. The SHR can be used to evaluate the strain-hardening effect of the material with load. The larger the SHR, the more obvious the strain-hardening effect during the process of plastic deformation. According to Figure 8, Mo-L, Mo-H, and Mo-C experience little strain hardening, and their sensitivities to the strain-hardening effect are different. As shown in Figure 9b, the sensitivities of the three molybdenum materials to the strain-hardening effect are ordered as follows: Mo-L > Mo-C > Mo-H.

### 4.4. Sensitivity to Temperature

To study how the dynamic yield strength varies with temperature, we use the dimensionless dynamic yield strength $\sigma^{*}$ and dimensionless temperature $T^{*}$, as shown in Formulas (6) and (7), where $T_{r}$ is the room temperature, $T_{m}$ is the melting point of the molybdenum material (2895 K), and T is the test temperature; $\dot{\epsilon^{*}}$, $C_{d}$, and $A_{d}$ are defined the same as in Formula (4).

(6) $$\sigma^{*}=\frac{\sigma }{C_{d}\ln\dot{\epsilon^{*}}+A_{d}}$$

(7) $$T^{*}=\frac{T-T_{r}}{T_{m}-T_{r}}$$

We study the relationship between the dynamic yield stress $\sigma^{*}$ and the dimensionless temperature $T^{*}$ using Formula (8).

(8) $$\ln\left(1-\sigma^{*}\right)=m\ln T^{*}+\ln E_{d}$$

The dynamic yield strength at different temperatures is regarded as the object of study. For the test results, see Table 3.

**Figure 10:**
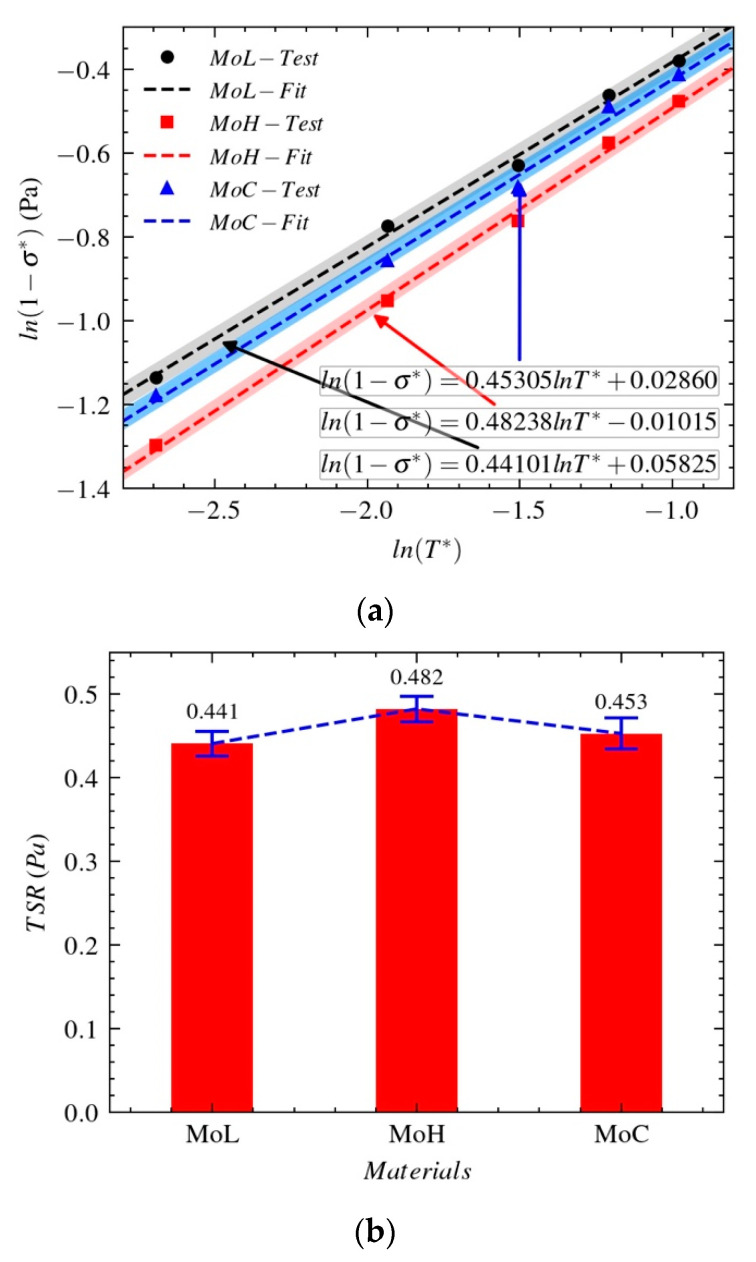
Sensitivities of the three molybdenum materials to temperature: (**a**) stress–temperature relationships (**b**) temperature-softening ratio.

With the model built using Formula (8), we study the relationships between the yield strength $\sigma^{*}$ and the dimensionless temperature $T^{*}$ of Mo-L, Mo-H, and Mo-C at different temperatures and strain rates. For the test results and model fitting result, see Figure 10a.

The temperature-softening ratio (TSR) in Figure 10b is obtained by determining the slope of the relationship between the true yield strength and temperature. The TSR can be used to evaluate the temperature-softening effect of the material with load. The larger the TSR, the more obvious the temperature-softening effect during the process of dynamic deformation. As shown in Figure 10b, the sensitivities of the three molybdenum materials to the temperature-softening effect are ordered as follows: Mo-H > Mo-C > Mo-L.

### 4.5. Modified Johnson–Cook Constitutive Model

Under different loading conditions, the strain-hardening effect, strain rate-strengthening effect, and temperature-softening effect of molybdenum materials during the process of deformation can be quantified through constitutive models. There are many constitutive models that describe the response of materials under different loading conditions. The Johnson–Cook (JC) [49,50] constitutive model is widely applied to assess the dynamic response of materials. This model can present the mechanical characteristics of a materials’ dynamic response instead of the microscopic properties. It uses multiplication to build a constitutive model. However, the JC constitutive model was first constructed for face-centred cubic (FCC) metals, which show evident hardening effects during the process of deformation. Therefore, the JC stress–strain curve described by the constitutive model shows that the stress changes divergently with strain [49,50]. Unlike FCC metals such as Cu, the strain-hardening effect of body-centred cubic (BCC) metals such as molybdenum is not significantly affected by temperature or strain rate [35,37]. This paper builds a modified JC (MJC) constitutive model, as shown in Formula (9), where A, B, C, D, E, n, and m are to be determined, and the meanings of the other parameters are the same as above.

(9) $$\sigma =\left(A+B\epsilon_{p}^{n}\right)\left(C+D\ln\dot{\epsilon^{*}}\right)\left[1-E{\left(\frac{T-T_{r}}{T_{m}-T_{r}}\right)}^{m}\right]$$

When $\epsilon_{p}=0$ and $T=T_{r}$, Formula (9) can be transformed as follows:

(10) $$\sigma =AC+ADln\dot{\epsilon^{*}}.$$

When $\epsilon^{*}=1$ and $T=T_{r}$, Formula (9) can be transformed as follows:

(11) $$\ln\left(\sigma -AC\right)=n\ln\epsilon_{p}+\ln\left(BC\right).$$

When $\epsilon_{p}=0$ and $\epsilon^{*}=1$, Formula (9) can be transformed as follows:

(12) $$ln\left(1-\frac{\sigma }{AC}\right)=m\ln\left(\frac{T-T_{r}}{T_{m}-T_{r}}\right)+\ln E.$$

**Figure 11:**
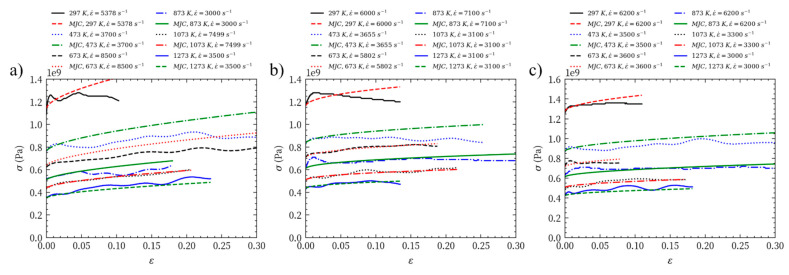
Comparison of true stress–strain curves evaluated from tests and fitted by MJC: (**a**) Mo-L; (**b**) Mo-H; (**c**) Mo-C.

By comparing Formulas (4), (5), (8), and (10)–(12), we know that $AD=C_{d}$, $AC=A_{d}$*,* $BC=B_{d}$, and $E=E_{d}$; i.e., we have five unknowns and four equations. With any other additional equation, we can determine parameters in the constitutive model of Formula (9). If C is a constant, based on the fitting results in Figure 7a, Figure 9a and Figure 10a, the MJC constitutive parameters of the three molybdenum materials can be determined, as shown in Table 4. For a comparison between the true stress–strain curve fitted by the MJC constitutive model at different temperatures and strain rates and the test results, see Figure 11.

Figure 11 shows that at temperatures of 873 K and above, the fitting curves of the MJC constitutive model for Mo-L, Mo-H, and Mo-C are almost identical to the test results. At temperatures ranging from 297 to 673 K, the fitted flow stress of MJC is higher than the true flow stress in the test. Particularly at 297 K and 473 K, the true flow stresses of Mo-L and Mo-H are significantly lower than the fitting result of the MJC constitutive model. This means that the mechanical response of molybdenum materials has a more complicated relationship with the temperature and strain rate under dynamic impact.

### 4.6. Rate-Temperature Equivalence

The strain rate effect and temperature effect of metal materials under dynamic impact are closely related to their mechanical properties. Generally, when the strain rate increases or the temperature decreases, the material hardens or embrittles [51,52,53]. In addition, through the quantitative analysis on the strain rate, temperature, and plastic strain of molybdenum materials under impact, as shown in Figure 11, we can see that the combined effect of the strain rate and temperature plays a key role in the mechanical response of molybdenum under impact load. We comparatively study the sensitivities of Mo-L, Mo-H, and Mo-C to the combined effect of strain rate and temperature under impact load and use the combined parameter $T_{t}^{*}$ of the equivalent strain rate $\epsilon^{*}$ and temperature T, as shown in Formula (13). For the assessment model of the relationship between flow stress σ and combined parameter $T_{t}^{*}$, see Formula (14).

(13) $$T_{t}^{*}=Tln\frac{\dot{\epsilon_{0}}}{\dot{\epsilon }}$$

(14) $$\sigma =A_{t}+B_{t}T_{t}^{*}+C_{t}{\left(T_{t}^{*}\right)}^{2}\;$$

**Figure 12:**
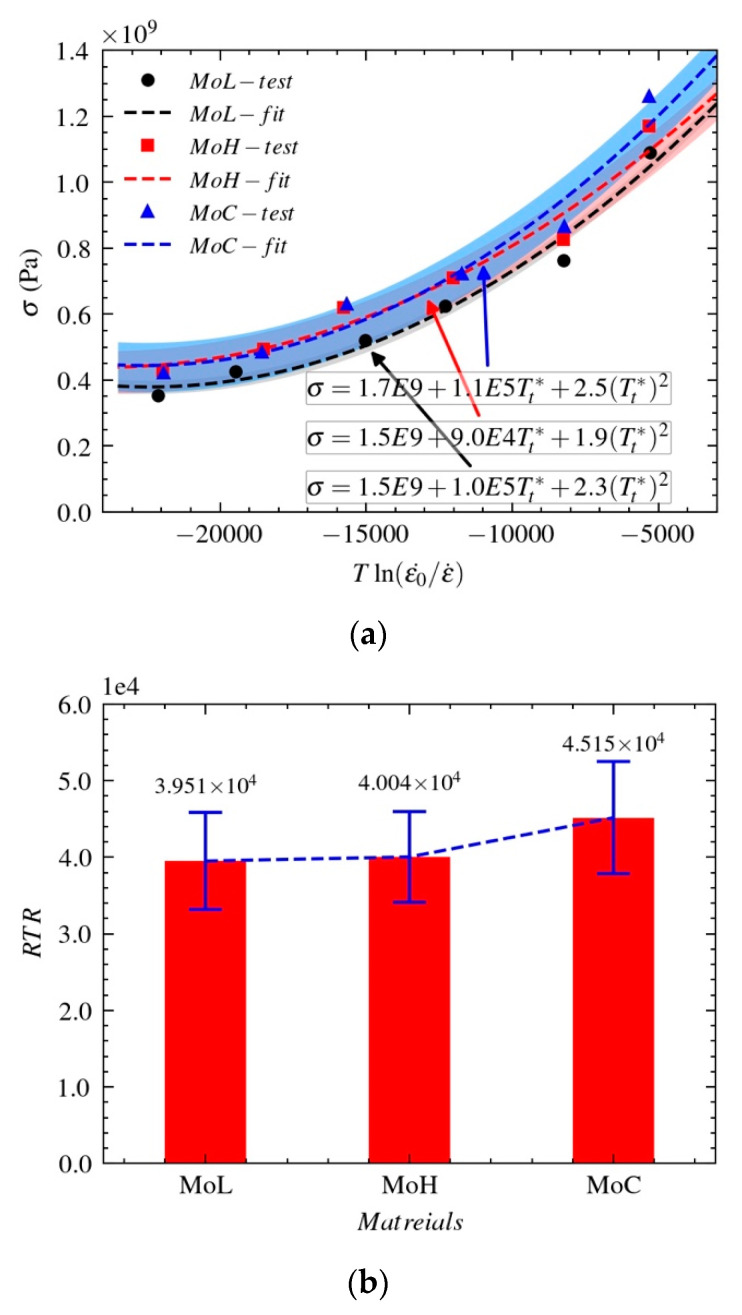
Relationship between the flow stress and the combined effect of the strain rate and temperature: (**a**) combined effect relationships (**b**) sensitivity ratio of combined effect.

Formula (14) fits the relationships between the flow stresses of the three molybdenum materials and the combined parameter $T_{t}^{*}$. A comparison between the test data and the fitted curves when $\epsilon_{p}=0$ is shown in Figure 12a. For the relationship between the flow stress and the combined parameter $T_{t}^{*}$ when $\epsilon_{p}=0$ is fitted with a linear relationship and the assessment of sensitivity to the combined effect of strain rate and temperature (RTR) through the slope of linear fitting, see Figure 12b.

As shown in Figure 12a, the curve fitted by the assessment model for the relationship between the flow stress σ and combined parameter $T_{t}^{*}$ is very consistent with the test data. This model can assess how the flow stress of molybdenum materials varies with the combined effect of strain rate and temperature under impact. The RTR in Figure 12b shows that Mo-L and Mo-H have almost the same sensitivity to the combined effect of strain rate and temperature, which is lower than that of Mo-C.

### 4.7. Temperature Modified Flow Stress

Molybdenum materials produce a large amount of deformation work that cannot be released during the process of high-speed deformation. Under impact at a high strain rate, the entire dynamic response becomes an adiabatic process, leading to adiabatic temperature increase. Formula (15) shows how the adiabatic temperature increase varies with stress–strain during the process of dynamic deformation.

(15) $$T=T_{0}+\frac{k\eta }{\rho C_{p}}\int_{0}^{\epsilon_{p}}\sigma d\epsilon_{p}$$

In Formula (15), k is the conversion coefficient of work and heat and is generally 0.95 [54] (<1) and η is the proportionality coefficient of the actual temperature increase and adiabatic temperature increase and ranges from 0 to 1 depending on the temperature and strain rate during the process of deformation [55]. At high strain rate, η=1, and $C_{p}$ is the nonlinear specific heat capacity. Formula (16) [56] shows how $C_{p}$ ($J\cdot {\mathrm{mol}}^{-1}\cdot K^{-1}$) of molybdenum metal varies with temperature (K) within the range of 500–2890 K.

(16) $$C_{p}=25.0575-0.0013T+3.491\times 10^{-6}T^{2}$$

By substituting Formula (16) into Formula (15) for mathematical transformation, we can obtain the adiabatic temperature increase equation under dynamic impact, Formula (17), where $A_{1}=25.0575$, $B_{1}=-0.0013$, and $C_{1}=3.491\times 10^{-6}$.

(17) $$T=T_{0}+\frac{k\eta \int_{0}^{\epsilon_{p}}\sigma d\epsilon_{p}}{\rho \left(A_{1}+B_{1}T+C_{1}T^{2}\right)}$$

After simplifying Formula (17), we can obtain the general form of the cubic temperature increase equation during the adiabatic process under impact. As shown in Formula (18), the real solution is the theoretical temperature of the material at any initial temperature $T_{0}$ when the plastic deformation of different flow stress σ reaches the plastic strain $\epsilon_{p}$.

(18) $$\rho C_{1}T^{3}+\left(\rho B_{1}-\rho C_{1}T_{0}\right)T^{2}+\left(\rho A_{1}-\rho B_{1}T_{0}\right)T-\rho A_{1}T_{0}-k\eta \int_{0}^{\epsilon_{p}}\sigma d\epsilon_{p}=0$$

**Figure 13:**
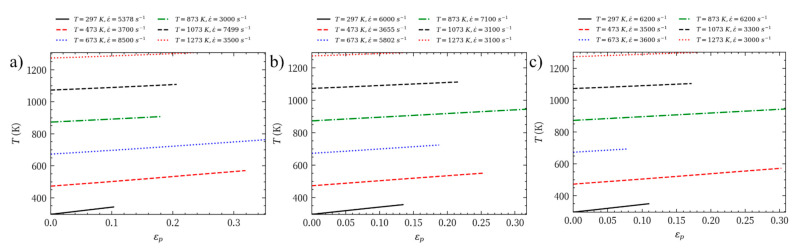
Adiabatic temperature increases of the three molybdenum materials: (**a**) Mo-L; (**b**) Mo-H; (**c**) Mo-C.

According to Formula (18), we know the relationship between the internal temperature of Mo-L, Mo-H, and Mo-C and the plastic strain under different temperatures and strain rates. The calculation results are shown in Figure 13. The results shows that the lower the initial temperature, the greater the adiabatic temperature increase during the process of plastic deformation, and that the greater the plastic deformation is, the higher the adiabatic temperature increase of the material; however, during the process of dynamic impact, the initial ambient temperature has a greater impact on the temperature inside the material.

**Figure 14:**
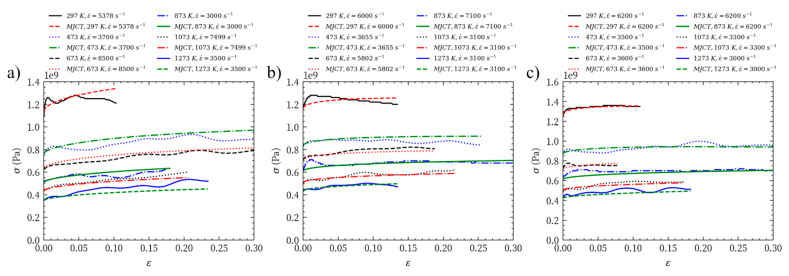
True stress–strain relationships from tests and MJC fitting curves: (**a**) Mo-L; (**b**) Mo-H; (**c**) Mo-C.

The flow stress σ and combined parameter $T_{t}^{*}$ are used to study the effect of the adiabatic temperature increases of the three molybdenum materials on the flow stress at different strain rates. According to the equation of the combined effect of the strain rate and temperature Formula (14), we know that the stress softening caused by adiabatic temperature increase is due to plastic deformation, as shown in Formula (19). The fitting result of the MJC constitutive model is modified based on the adiabatic softening model of Formula (19). For a comparison between the modified true stress–strain relationships of Mo-L, Mo-H, and Mo-C under dynamic compression and the MJC fitting result, see Figure 14.

(19) $$\Delta \sigma =B_{t}\Delta T_{t}^{*}+C_{t}{\left(\Delta T_{t}^{*}\right)}^{2}$$

The stress softening Δσ caused by the adiabatic process is introduced to modify the MJC fitting result. By comparing how the flow stresses of Mo-L, Mo-H, and Mo-C vary with plastic strain, we know that at high temperature, the MJC results are consistent with the test data. At room temperature, the fitting curve of MJC is consistent with the test results of Mo-C, while the flow stresses of Mo-L and Mo-H are higher than the test values. The difference between MJC and test results is the lower deformation resistance caused by the response of microstructure when Mo-L and Mo-H resist dynamic impact. Further research into the response of the microstructures of Mo-L and Mo-H under high-speed impact may help us better understand the abnormal difference in the flow stresses of molybdenum materials under dynamic impact at room temperature. In summary, the MJC description of the plastic flow behaviour of molybdenum materials at high temperature and high strain rate is almost the same as the test result, while its description of the flow stress of BCC metals such as molybdenum is better than the JC constitutive model.

According to the comparative study of Mo-L, Mo-H, and Mo-C, there are no significant differences between highly pure Mo-L and Mo-H and common commercial Mo-C in terms of the sensitivity to the temperature-softening effect or strain rate-strengthening effect during the process of impact. Different processing deformations of high-purity molybdenum have different effects on the strain rate or temperature effect of the material during the process of impact. A large amount of processing deformation increases the sensitivity of materials to temperature, while a small amount of processing deformation increases the sensitivity of materials to strain rate. However, the analysis of the combined effect of strain rate and temperature indicates that high-purity molybdenum materials respond almost the same. The sensitivities of Mo-L and Mo-H to the combined effect of strain rate and temperature are significantly lower than that of Mo-C and irrelevant to the processing deformation of high-purity molybdenum. Therefore, improving the purity of molybdenum materials can reduce their sensitivity to the combined effect of strain rate and temperature under high-speed impact. Different processing deformations have different effects on the temperature-softening effect or strain rate-strengthening effect during the process of impact.

The strain-strengthening effect during the process of deformation is not closely related to the purity of the material but is strongly related to the amount of deformation during the processing of the material. The smaller amount of processing deformation, the more obvious the strain strengthening effect during the deformation of the material.

### 4.8. Impact Embrittlement and Toughening

During the process of the dynamic impact of molybdenum materials, adiabatic softening is one of the factors causing plastic flow stress unloading. In addition, defects such as cracks inside the material may have an impact on plastic flow stress unloading. Figure 14 shows that while the fitting results of the MJC constitutive model are consistent with the test results at high temperature and high strain rate, the relationship between the flow stress and the plastic strain of molybdenum materials under impact load at room temperature fitted by the MJC constitutive model is different from the test data. By observing microstructures of molybdenum materials after impact at room temperature and high temperature, as shown in Figure 3, Figure 4 and Figure 5, we find that molybdenum materials are prone to producing a large number of structural defects such as cracks at room temperature and high strain rate, although this phenomenon is not evident at high temperature. Researching the difference in the micro-failure mechanisms of molybdenum materials under the impact load of high temperature and room temperature is significant for understanding the mechanical response of molybdenum materials at room temperature.

**Figure 15:**
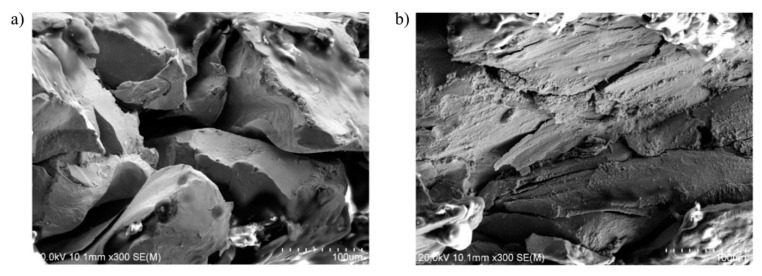
Fractures of Mo-H under SEM: (**a**) test temperature 297 K, $\dot{\epsilon }=11,000\;s^{-1}$; (**b**) test temperature 1273 K, $\dot{\epsilon }=6289{\;s}^{-1}$.

By taking Mo-H as an example, we study the mechanism of producing cracks in molybdenum materials under impact. The metallographic microstructure at temperature 297 K and strain rate $11,000{\;s}^{-1}$ is shown in Figure 4a. There are a large number of irregular cracks generated inside Mo-H. The information about the fracture surface of Mo-H obtained through scanning electron microscopy (SEM) is shown in Figure 15a. At high strain rate and room temperature, most of the cracks generated during the dynamic response of molybdenum materials are grain-boundary fractures. The metallographic microstructure under impact load at 1273 K and under a strain rate of $6289{\;s}^{-1}$ is shown in Figure 4b. There are fewer cracks inside the materials. The information about the crack section of Mo-H obtained through SEM is shown in Figure 15b. The cracks are mainly generated as a result of cleavage fracture inside the crystal. According to analysis of the fracture mechanism at room temperature and high temperature, molybdenum materials are easy to embrittle at room temperature and high strain rate, causing a large number of internal microstructures inside the material to be damaged and affecting the plastic performance of the material. However, once the temperature of the impact environment increases, the internal damage of the material changes from intergranular fracture to cleavage fracture, the number of microdefects remarkably decreases, and the molybdenum materials show significant improvement in plastic properties. The higher the temperature, the better the plastic properties and impact toughness of molybdenum materials are.

By comparing the microstructures of Mo-L, Mo-H, and Mo-C before and after the impact, we know that at room temperature (297 K), Mo-H, with more processing deformation, shows many microdefects, while Mo-L, with less processing deformation, has fewer microdefects. Compared to the commercial Mo-C, the high-purity molybdenum with less processing deformation has better plastic properties during the process of impact. At high temperature (1273 K), the plastic properties of the three molybdenum materials all improve. In particular, those of Mo-L are better than those of Mo-H and Mo-C.

## 5. Conclusions

We studied the plastic behaviour and failure behaviour of three molybdenum materials at different temperatures and high strain rates. The conclusions are given below:

The strain rate sensitivity (SRR), strain sensitivity (SHR), and temperature sensitivity (TSR) of molybdenum materials were constructed to quantitatively analyse the response behaviour of molybdenum materials under high-temperature and high-strain-rate impacts. The sensitivities of the high-purity materials to the combined effect of strain rate and temperature are consistent, and the materials with higher purity are less sensitive to the combined effect of strain rate and temperature. The strain-strengthening effect is mainly related to the amount of processing deformation; the smaller the amount of processing deformation, the more pronounced the strain-strengthening effect during the deformation process.

Pure molybdenum materials are prone to embrittlement at room temperature and high strain rate, which affects the plastic performance of the material. However, when the temperature of the impact environment increases, the internal damage of the material changes from intergranular fracture to cleavage fracture, the number of microdefects remarkably decreases, and the dynamic plastic properties of the molybdenum materials improve.

A Modified Johnson–Cook constitutive model is established. Based on the model for the combined effect of strain rate and temperature and the modified model for temperature and flow stress during the adiabatic process under impact, we assess the complicated mechanical response of molybdenum materials with different purities and processing deformation conditions under dynamic impact. The calculation results are consistent with the test results. The research results are helpful for better understanding the complicated dynamic plastic behaviour of molybdenum materials and making full use of the plastic properties of molybdenum materials as needed.

## Figures and Tables

**Table 1:** Compositions of the three molybdenum materials.

Abbreviation	Mo%	Contents of Impurity Elements			
		Al	Si	C	O
Mo-L	≥99.99	2	10	20	22
Mo-H	≥99.99	2	10	20	22
Mo-C	≥99.95	3	14	25	28

**Table 2:** Yield strengths at different strain rates.

Material		Mo-L		Mo-H		Mo-C	
Test	Temperature	Strain Rate	Yield Strength	Strain Rate	Yield Strength	Strain Rate	Yield Strength
Test 1	297 K	0.0001	411 MPa	0.0003	498 MPa	0.0003	599 MPa
Test 2	297 K	\	\	0.003	577 MPa	0.003	665 MPa
Test 3	297 K	0.01	629 MPa	0.03	725 MPa	0.03	825 MPa
Test 4	297 K	5378	1135 MPa	6000	1149 MPa	6200	1267 MPa

**Table 3:** Yield strengths at different temperatures and strain rates.

Material		Mo-L		Mo-H		Mo-C	
Test	Temperature	Strain Rate	Yield Strength	Strain Rate	Yield Strength	Strain Rate	Yield Strength
Test 1	273 K	1151	942 MPa	6000	1170 MPa	6200	1260 MPa
Test 2	473 K	3700	764 MPa	3655	826 MPa	3500	866 MPa
Test 3	673 K	8500	624 MPa	5802	709 MPa	3600	721 MPa
Test 4	873 K	3000	521 MPa	7100	620 MPa	6200	630 MPa
Test 5	1073 K	7499	427 MPa	3100	494 MPa	3300	484 MPa
Test 6	1273 K	3500	354 MPa	3100	428 MPa	3000	422 MPa

**Table 4:** MJC constitutive parameters.

	A	B	n	C	D	E	m
Mo-L	4.25E8	3.98E8	0.60	1	0.0946	1.060	0.441
Mo-H	4.66E8	1.76E8	0.45	1	0.0826	0.990	0.482
Mo-C	5.59E8	2.23E8	0.51	1	0.0712	1.029	0.453

## Data Availability

The data presented in this study are available on request from the corresponding author.
